# Multimodal Approach for Pilot Mental State Detection Based on EEG

**DOI:** 10.3390/s23177350

**Published:** 2023-08-23

**Authors:** Ibrahim Alreshidi, Irene Moulitsas, Karl W. Jenkins

**Affiliations:** 1 Centre for Computational Engineering Sciences, Cranfield University, Cranfield MK43 0AL, UK; 2 Machine Learning and Data Analytics Laboratory, Digital Aviation Research and Technology Centre (DARTeC), Cranfield University, Bedford MK43 0AL, UK; 3 College of Computer Science and Engineering, University of Ha’il, Ha’il 81451, Saudi Arabia

**Keywords:** ensemble learning, machine learning, EEG, pilot deficiencies, artifact detection, tangent space, EEG preprocessing, heterogeneous data, mental states classification, feature extraction

## Abstract

The safety of flight operations depends on the cognitive abilities of pilots. In recent years, there has been growing concern about potential accidents caused by the declining mental states of pilots. We have developed a novel multimodal approach for mental state detection in pilots using electroencephalography (EEG) signals. Our approach includes an advanced automated preprocessing pipeline to remove artefacts from the EEG data, a feature extraction method based on Riemannian geometry analysis of the cleaned EEG data, and a hybrid ensemble learning technique that combines the results of several machine learning classifiers. The proposed approach provides improved accuracy compared to existing methods, achieving an accuracy of 86% when tested on cleaned EEG data. The EEG dataset was collected from 18 pilots who participated in flight experiments and publicly released at NASA’s open portal. This study presents a reliable and efficient solution for detecting mental states in pilots and highlights the potential of EEG signals and ensemble learning algorithms in developing cognitive cockpit systems. The use of an automated preprocessing pipeline, feature extraction method based on Riemannian geometry analysis, and hybrid ensemble learning technique set this work apart from previous efforts in the field and demonstrates the innovative nature of the proposed approach.

## 1. Introduction

The evolution of the aviation industry is heavily dependent on maintaining the highest standards of safety. Advances in aircraft design, endurance, and safety have led to a decrease in the number of aircraft accidents worldwide since the 1960s [1]. However, operator reliability remains a crucial factor in maintaining flight safety, as flight crews are responsible for a wide range of tasks, including receiving instructions from air traffic control, interpreting onboard instrument data, making course corrections, briefing cabin crew and passengers, and responding to unexpected events. Operating an airplane requires a high level of mental acuity, and these responsibilities can compromise flight safety [2,3,4]. According to data analyzed by the International Air Transport Association (IATA), there were 45 plane crashes caused by pilots losing control of the aircraft, resulting in 1645 fatalities between 2012 and 2021 [5,6]. Furthermore, the Commercial Aviation Safety Team (CAST) investigated 18 aircraft accidents in which pilots lost control and found that deficiencies in flight crew attention were involved in 16 of the 18 incidents [7]. As a result, CAST recommended that the aviation community, which includes government, business, and academic institutions, conduct research to detect and assess attention-related pilot performance deficiencies (APPD), specifically focusing on channelized attention (CA), diverted attention (DA), and startle/surprise (SS) mental states. CA is a state where pilots engage in a puzzle-based video game called Tetris while remaining focused entirely on the game without paying attention to other tasks. DA is a state in which pilots solve math problems that periodically appear while performing display monitoring tasks. Pilots who are in the SS mental state experience unexpected inversions of the primary flight display in the simulator.

To achieve this goal, researchers from both academia and industry have investigated a variety of approaches based on physiological signals and machine learning (ML) methods. In terms of physiological signals, quantitative sensors, both singular and multiple, have been employed to capture biological signals from the human body in both field studies and near-realistic laboratory environments. The electroencephalography (EEG) sensor is widely regarded as the most crucial physiological signal for analyzing mental states due to its ability to detect transient alterations in brain activity that may be indicative of pilots’ attention deficits. It seems to provide the most accurate data for distinguishing mental states. It is also preferable to other methods of brain monitoring since it is safe, adaptable, non-invasive, and an utterly passive recording technique. Despite its advantages, EEG is notorious for picking up artefacts from environmental factors and physiological phenomena, such as muscle activity, ocular movements, line noise, and heartbeats, which compromise the quality of the signals. Therefore, isolating the neural signal relative to the cognitive processes that reflect brain activity from the recorded artefacts is crucial.

The presence of artefacts in EEG data can negatively impact the performance of ML models used to detect different mental states of pilots. To address this issue, researchers have employed various signal processing and feature extraction techniques. One approach is to record and combine EEG with non-brain physiological signals, such as functional near-infrared spectroscopy, electrocardiogram (ECG), galvanic skin response (GSR), and respiration (RP), simultaneously. However, the fusion of features derived from EEG and non-brain physiological signals may not always improve the performance of ML models [8,9]. Another approach is to utilize traditional preprocessing techniques to handle contaminated EEG data. Visual inspection and rejection, filtering, and Independent Component Analysis (ICA) are examples of such conventional denoising procedures. These methods, while effective, have several downsides, including the need for manual implementation, being slow and inefficient for longer recording sessions, and being difficult for beginners to execute [10,11]. These drawbacks highlight the importance of developing an automated preprocessing method.

Features or essential information embedded in the EEG signal are usually extracted after preprocessing, as they are crucial for classification tasks [12,13,14]. Both temporal and spatial features can be extracted from the EEG signals. For pilot mental state classification, temporal features in the time, frequency, and time–frequency domains are commonly extracted [15]. One such method that originates in the frequency domain is the power spectrum density (PSD). The presence or absence of shifts in the power spectra of individual EEG bands is an important indication of different mental states. In brain–computer interface (BCI) applications, spatial features are commonly extracted. They represent the active area of the brain. For pilot mental state classification, they are rarely used as input.

Features extracted from EEG signals are then fed into an ML model to predict various types of mental states. ML models are trained to distinguish between either binary or multiple classes. Fatigue, workload, stress, and drowsiness are examples of detected mental states in the literature. Most studies have attempted to establish a clear distinction between normal (NE) and each mental state (i.e., a binary classification) or to categorize a single mental state into three or more distinct levels. In addition, only a few studies have focused on assessing and detecting attention-related pilot performance deficiencies (APPD). To the best of our knowledge, no attempts have been made to simultaneously recognize different APPD states (i.e., multiclass classification), particularly CA, DA, SS, and NE, using solely EEG data.

This study aims to investigate the viability of identifying APPD states using publicly released EEG data. Specifically, the study poses the following research questions: (1) Can an advanced automated EEG preprocessing pipeline be developed to clean the dataset? (2) Can spatial features that are relevant to predicting pilot mental states, such as CA, DA, and SS, be extracted from cleaned EEG data? (3) Can a hybrid ensemble learning model be developed to classify four pilot mental states based on heterogeneous EEG data using spatial features? (4) Will the hybrid ensemble learning model outperform other ML models? (5) How can the results of this study contribute to the development of tools and techniques for detecting and assessing attention-related pilot performance limitations/deficiencies in aviation settings?

In this work, we propose a novel multimodal approach that decontaminates the EEG signals, extracts meaningful features, and detects the APPD states using heterogeneous cleaned EEG signals collected from 18 pilots. The main contributions of this paper are as follows:

- Development of automatic preprocessing pipeline to automatically repair or remove corrupted EEG data.
- Development of feature extraction and selection methodology, based on Riemannian geometry analysis of the cleaned EEG data, that handles the issues of an imbalanced dataset and the curse of dimensionality and extracts meaningful features from the EEG signals.
- Development of a novel APPD system based hybrid ensemble learning for classifying CA, DA, SS, and NE states.

Recognition of APPD mental states was critically examined using several different ensemble learning algorithms, including Random Forests (RF), Extremely Randomized Trees (ERT), Gradient Tree Boosting (GTB), AdaBoost, and hybrid ensemble learning (Voting). By addressing these research questions and providing these contributions, this study provides new insights into the use of EEG data to predict and assess APPD, as recommended by the CAST.

The remaining sections of this work are structured as follows: In Section 2, we briefly examine relevant works. The existing EEG recordings, the proposed multimodal approach, and the proposed ML classification models are described in Section 3. In Section 4, we report and discuss experimental findings. Section 5 wraps up the investigation and suggests some directions to explore next in terms of research.

## 2. Related Work

The process of identifying mental states typically involves four steps: collecting data, cleaning it, selecting relevant features, and making predictions. The first step involves capturing signals from the brain and converting them into digital form. Then, to ensure accurate analysis, any extraneous noise or artifacts present in the data are removed through preprocessing. Next, specific characteristics of the data are selected and extracted in preparation for classification. These extracted features are then used by a classifier to make predictions about which class the data belongs to. As this process specifically relates to EEG data, the following provides a summary of previous research on the three stages of mental state detection: preprocessing, feature extraction, and classification.

### 2.1. Signals Preprocessing

An assortment of neuronal activity, physiological artefacts, and non-physiological noise can be found in raw EEG data. As their presence may hinder the performance of ML models [16], identifying and removing artefacts is a crucial preprocessing step before their use [17]. Although most research preprocessed their EEG data, there were a few exceptions [18,19,20]. To increase the signal-to-noise ratio (SNR), it is necessary to undertake a preprocessing procedure to eliminate extraneous noise and artefacts.

For the pilot’s mental states classification, conventional preprocessing techniques, including filtering [16,21,22,23,24,25,26,27] and ICA [24,25,28], were employed on the EEG recordings. For example, Roza et al. [16] used a band-pass filter with a center frequency of 12–30 Hz to isolate the beta rhythm. Han et al. [25] used band-pass filtering at 0.1–50 Hz to remove the high frequency prior to removing eyes-related artefacts using the ICA algorithm. Similarly, Alreshidi et al. [29] used previously released pilot EEG data to analyze the influence of three preprocessing procedures on the efficiency of two ML models. The results demonstrated no discernible changes in the performance accuracies of the models when the data were filtered or when ICA was applied for eye-related artefact detection after data filtration. It has been established in the literature that typical preprocessing procedures for EEG data analysis necessitate knowledge and experience on the part of the analyst. Furthermore, they are only applicable when applied manually, requiring inspection, identification, and removal of faulty channels and contaminated data segments.

The past few years have seen the development of a number of partially or completely automated EEG preprocessing procedures that provide ways to clean EEG data. The Autoreject algorithm is an example of an automated preprocessing procedure that can be employed in EEG analysis pipelines [30]. It is a novel approach for automatically identifying and repairing erroneous segments in EEG data from single trials. It uses advanced statistical learning techniques, such as Bayesian hyperparameter optimization and cross-validation, to select amplitude thresholds to use for rejecting or repairing bad segments in EEG data. The Autoreject technique was used by Bonassi et al. [31] to automatically repair or reject contaminated epochs in EEG data. Pousson et al. [32] preprocessed the EEG data that were recorded from pianists doing musical tasks using the Autoreject method. There was a total of 10% erroneous epochs that were uncovered by the method and subsequently omitted from the investigation. Previous research has established that Autoreject has a significant role in the automatic purification of EEG data.

### 2.2. Feature Extraction

EEG is a set of stochastic signals that conceals extremely intricate data. Because of its high nonlinearity, its features are prone to sudden fluctuations. Human mental states, however, transition gradually from one state to the next [33]. Feature extraction aims to extract relevant features from data to map EEG segments to mental states.

Various features, such as statistical [16,22,34] and power spectral density features [16,18,21,22,23,24,25,28,34,35], have been extracted from pilots’ EEG recordings in earlier research in order to classify pilots’ mental states. For example, Wu et al. [28] used the power spectrum curve area representation of the decomposed delta, theta, alpha, and beta brain waves obtained using wavelet packet transform as features to perform the classification. Roza et al. [16] derived 15 distinct features from EEG and other physiological signals. The wavelet coefficients and several statistical features were extracted from the EEG signals. Furthermore, Binias et al. [26] extracted logarithmic band-power features using common spatial pattern (CSP) spatial filtering, which is widely used in BCI applications, from pilots’ EEG recordings.

There has been a recent uptick in the use of Riemannian geometry-based feature extraction and classification algorithms for BCIs. The first implementation of these techniques in BCI applications was published in [36]. The authors employed the Riemannian mean covariance matrix distance as a feature for classification purposes. Additionally, they showed how the covariance matrices can be represented as vectors in the tangent space of the Riemannian manifold. Majidov and Whangbo [37] computed the covariance matrices obtained by using CSP spatial filtering and mapped them onto the tangent space of the Riemannian manifold. Singh et al. [38] used the data from the EEG electrodes to create spatial filters that reduce the dimensionality prior to employing Riemannian distance as a pattern recognition metric for classification. In addition, classifiers based on Riemannian geometry were used by Appriou et al. [39] in the proposed BioPyC toolbox. One such classifier is the tangent space classifier.

### 2.3. Mental State Classification

After EEG signals have had their features extracted, they must be classified using either a binary or multiclass ML approach. Because of the increased efficiency with which neural data may be analyzed and the need to decode brain activity, ML, and particularly Deep Learning (DL), algorithms have found widespread use in the field of computational neuroscience. Supervised ML algorithms, for instance, must first be trained using example data. The model and its learnt properties are then used to make predictions about the class label of new data that have not yet been seen.

For the detection of various pilot mental states, previous studies implemented various ML [18,22,23,24,25,26,27,34,35,40,41] and DL [16,18,25,26,28,35,42,43] algorithms. For instance, Han et al. [25] proposed a detection system based on multimodal physiological signals and a multimodal deep learning (MDL) network, consisting of convolutional neural network (CNN) and long short-term memory (LSTM) algorithms, to detect pilot’s mental states, namely distraction, workload, fatigue, and normal. Roza et al. [16] proposed an emotion recognition system based on multimodal physiological signals and artificial neural network (ANN). The system was developed to detect five emotional states, namely happy, sad, angry, surprised, and scared. To identify the various states of mental fatigue, Wu et al. [28] presented a deep contractive autoencoder network; up to 91.67 percent of cases of the fatigued mental status of pilots could be correctly identified. In a flight simulator experiment, Johnson et al. [23] investigated probe-independent methods for categorization of three layers of task-complexity. The investigation was carried out using six classification algorithms, namely naïve bayes, decision trees, quadratic discriminant analysis, linear discriminant analysis (LDA), k-nearest neighbors (KNN), and support vector machine (SVM). Dehais et al. [40] devised a scenario in which twenty-two pilots using a six-dry-electrode EEG system performed a low-load and high-load traffic pattern, as well as a passive auditory oddball. Zhang and Wang [24] proposed a concatenated structure of deep recurrent and 3D CNN to learn spatial–spectral–temporal EEG features for cross-task mental workload assessment. The findings reveal that the proposed approach achieved an average accuracy of 88.9%. Distinguishing between stages of brain activity related to idle but concentrated anticipation of visual cues and reactions to them using LDA, KNN, SVM, RF, and ANN algorithms was the focus of the research of Binias et al. [26].

Detecting and assessing APPD was also addressed in previous studies. For example, Harrivel et al. [35] employed RF, extreme gradient boosting, and deep neural network classifiers to predict CA, DA, and low workload states. As a preliminary study, through the use of different sensing modalities in high-fidelity flight simulators, the authors classified three types of mental states. Harrivel et al. [34] employed RF, gradient boosting, and two SVM classifiers to identify CA and SS states in further studies. The authors stressed the need for addressing the data quality issues. Terwilliger et al. [20] aggregated three mental states classes, namely CA, DA, and SS, into one class called event. To distinguish the event class from the NE mental state class, the authors presented a convolutional autoencoder approach. In previous research, we examined the effects of two preprocessing procedures on SVM and ANN using EEG data from a pilot exposed to CA, DA, SS, and NE states [29]. Although the models demonstrated the viability of combining data from two scenarios, the curse of dimensionality prevented them from accurately predicting the DA and SS states.

In the field of aviation, several studies have been conducted to evaluate the efficacy of EEG data in predicting mental states of pilots. Some of these studies have employed a binary classification approach to detect different mental states, while others have utilized EEG data in combination with other physiological data to improve performance. In this study, we develop a multiclass classification approach to identify CA, DA, SS, and NE states using only EEG data.

Another notable limitation of previous studies is the limited sample size, with many only incorporating EEG data from fewer than 10 participants. This raises questions regarding the generalizability of their results, as the findings may only be applicable to a small subset of the population. While incorporating additional signals can sometimes improve model performance, it can also introduce additional noise and complexity to the system, making it more challenging to interpret the results. In this work, we develop our model using only cleaned heterogeneous EEG data collected from 18 pilots, which provides more generalization.

Additionally, some studies have not disclosed the necessary information to make their work easily reproducible, while others have failed to make their datasets publicly available. This makes it challenging for other researchers to verify or build upon their findings. In this work, we train our models with publicly released EEG data, which makes it reproducible.

Furthermore, some studies have not performed proper preprocessing techniques on their EEG data, such as advanced filtering and artefact removal, potentially compromising the validity of their results. The noise can interfere with the extraction of meaningful features and patterns in the EEG signal, leading to a decrease in the accuracy and reliability of the resulting model. To minimize the impact of noise on the performance of ML techniques, it is important to preprocess the EEG signal and remove as much noise as possible before training the model. Accordingly, we develop an automated preprocessing pipeline in this study to automatically clean and improve the quality of the EEG signals.

Regarding extracting meaningful features for the machine learning models, researchers have hardly ventured beyond statistical and PSD features. In this work, we extract tangent space vectors based on Riemannian geometry analysis in an attempt to detect APPD states.

To the best of our knowledge, current research did not attempt to combine multiple approaches from different areas to predict the pilot’s mental states, which makes this study the first of its kind in the aviation field. The innovative nature of this study lies in the development of a novel multimodal approach to detect and classify APPD states using cleaned EEG data. The EEG signals from 18 pilots were collected from a variety of conditions to form the heterogeneous EEG data. The approach involves the automatic preprocessing of the EEG signals, feature extraction and selection methodology based on Riemannian geometry analysis, and a novel APPD system that classifies the APPD states. The system addresses the issues of corrupted EEG data, imbalanced datasets, and the curse of dimensionality, and provides meaningful features from the EEG signals, making it a unique contribution to the field.

## 3. Materials and Methods

### 3.1. Dataset Description

In November 2020, a dataset was obtained from NASA’s open data portal website, which comprised experimental data collected from 18 pilots. The pilots participated in four experiments, three of which took place in a non-flight environment and one in a high-fidelity motion-based flight simulator. The non-flight environment experiments lasted approximately 6 min, while the flight simulator experiment lasted approximately 1 h. The data were recorded in physiological signals and were provided in CSV format. Information regarding the utilized EEG recording headset and the flight simulator is reported in Appendix A.

**Figure 1:**
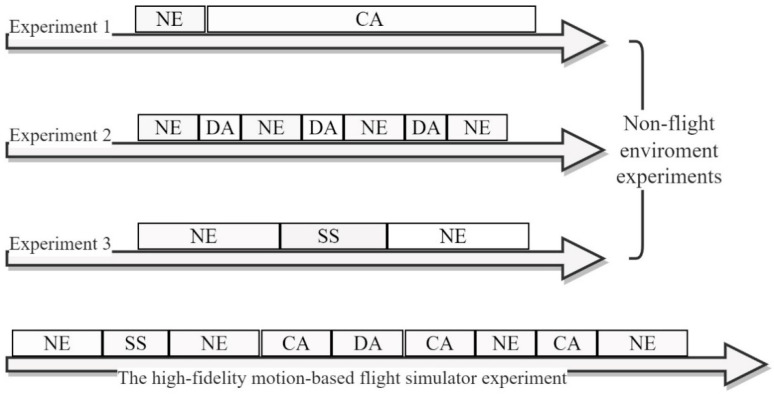
A typical snapshot and schematic of each experiment.

The dataset was divided into one-second epochs and combined into a single dataset of 89,198 samples, to account for the varying durations of each benchmark task. The benchmark tasks included NE, CA, DA, and SS. A typical snapshot and schematic of each experiment is depicted in Figure 1. The majority of the samples in the dataset came from the NE class (80%).

This dataset has great potential for advancing research in the fields of BCI and human factors in aviation and can be used to develop new models and algorithms to predict pilot performance under different conditions, as well as training programs to improve pilot performance in high-stress situations. Additionally, the dataset can be utilized to evaluate the design of flight deck interfaces and test the effectiveness of new technologies, such as augmented reality and virtual reality, in enhancing pilot performance.

### 3.2. The Automatic Preprocessing Pipeline

**Figure 2:**
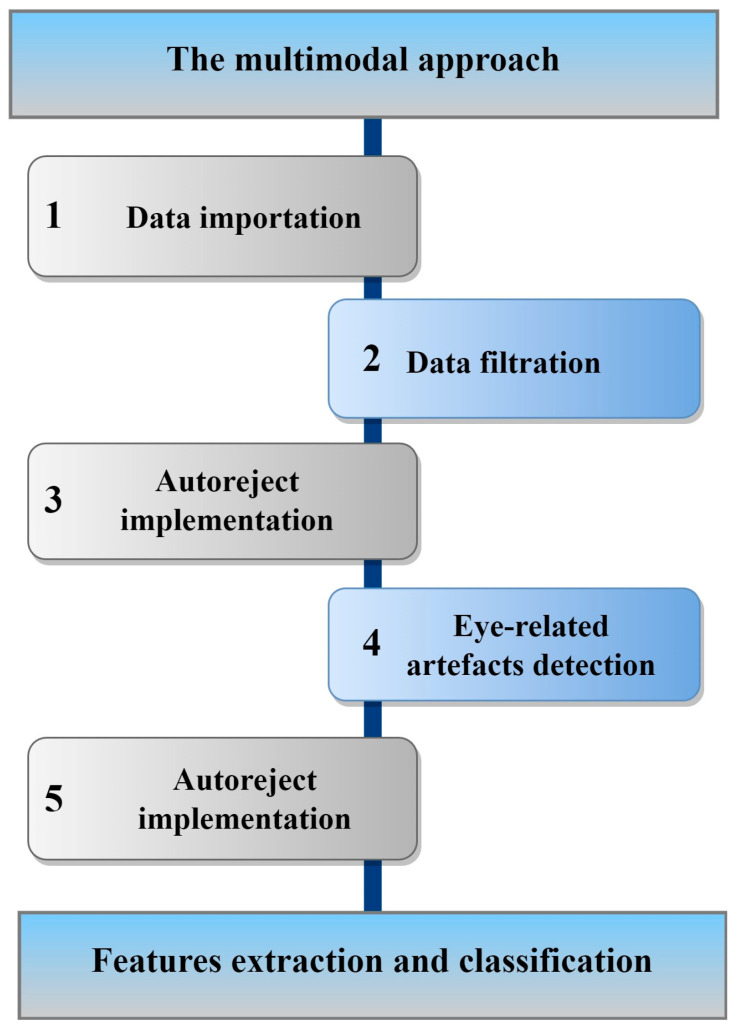
An outline of the multimodal approach based on EEG.

This study implemented advance preprocessing techniques using an open-source library called MNE-Python. The proposed EEG data preprocessing pipeline is shown in Figure 2. A brief description of the preprocessing steps is discussed below.

The EEG data were given in a CSV file. We used the MNE-Python library to apply advanced preprocessing methods. A “raw” object, a core data structure for continuous EEG data, was created and included information such as channel names and types, standard montage labeling, and the sample rate.

**Figure 3:**
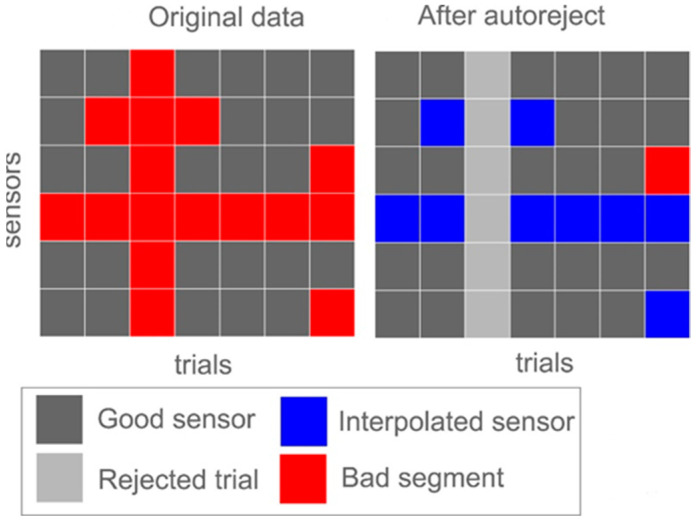
A simplified form of the Autoreject algorithm operation.

The first step was to filter the EEG signals. This was achieved by applying a digital filter to the data, which suppresses specific frequency components that fall outside of a designated range. There are two main types of digital filters used in digital signal processing (DSP): finite impulse response (FIR) and infinite impulse response (IIR). In the present study, we applied band-pass filtering to the EEG signals using an FIR filter, with a cutoff range of 1–50 Hz. We then segmented the EEG data into one-second non-overlapping epochs. The epochs that had a maximum peak-to-peak signal amplitude of more than 700 µV, or a minimum peak-to-peak signal amplitude of less than 1 µV, were dropped from the dataset, as their existence negatively affected the applicability of the next preprocessing steps. Afterwards, we employed the Autoreject method to repair or discard corrupted epochs. Bayesian optimization and cross-validation are leveraged in Autoreject to automatically determine an artefact threshold for each channel/sensor; thereafter, faulty channels/sensors are interpolated, or the epoch is discarded. Figure 3 is a diagram depicting the operation of the Autoreject algorithm in a simplified form. For a detailed discussion of how and why this algorithm works, we suggest reading [38], written by the program’s creators. To identify and eradicate blinks and other forms of artifactuality, we employed an MNE-Python function that used the EEG channel Fp1 as a surrogate electrooculogram. These components have a lot of variation and tend to be located in the frontotemporal region of the head. The EEG signals were reconstructed after the blinking component was eliminated from the source matrix. Finally, we used Autoreject again to encounter any distortions that could be found after repairing the blink artefacts.

With more than 80% of the data coming from the NE class, it is possible that the trained model will be biased toward that class. This makes a model’s predictions seem naive, even if they have a high degree of accuracy. To counteract the preponderance of the NE class, we undersampled the data with the intention of creating a more even distribution across all classes.

### 3.3. EEG Feature Extraction

**Figure 4:**
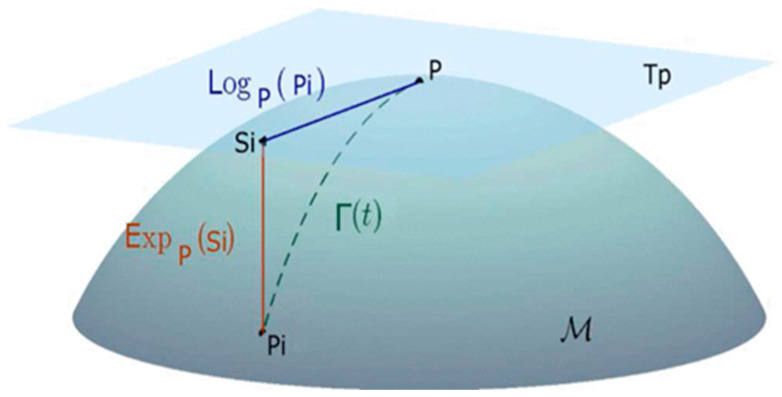
A geometric depiction of the tangent space mapping process.

After preprocessing the EEG data, two methods that expanded upon previous work on EEG BCI were adopted. First, the EEG data were subjected to specialized spatial filtering in order to boost SNR. We used an algorithm modified from the xDawn algorithm to estimate the spatial filters. Second, we extracted the features from a particular form of the EEG epochs’ covariance matrices and adjusted them using techniques from Riemannian geometry. Indeed, the covariance matrices, being Symmetric and Positive-Definite Matrices (SPD), are topologically localized on a Riemannian manifold. To reduce the covariance matrices dimensionality by discarding irrelevant information, we performed the Fisher Geodesic Discriminant Analysis (FGDA) algorithm proposed by [44,45]. Be aware that the features are matrices, rather than the typical vectors. Because we need to maintain the special structure of these matrices, we cannot simply vectorize them. As an alternative, we employed techniques from Riemannian geometry introduced in [46] to map the covariance matrices, belonging to a manifold, onto the Riemannian tangent space, where they may be vectorized and treated as Euclidean objects. Each matrix is represented as a vector of size *n*(*n* + 1)/2, where *n* is the dimension of the SPD matrices. Figure 4 is a geometric depiction of the tangent space mapping process. Despite its more common association with motor imagery, we believe that incorporating it into a visual processing task as part of our research could prove to be useful. A tangent space formed by a group of tangent vectors can be defined for each point P, where P ∈ P(n). Between P and the exponential mapping $P={Exp}_{p}(S_{i})$, each tangent vector S is the derivative at t=0 of the geodesic Γ(t), denoted as

(1) $${Exp}_{P}\left(S_{i}\right)=P^{\frac{1}{2}}\exp(P^{-\frac{1}{2}}S_{i}P^{-\frac{1}{2}})\;P^{\frac{1}{2}}$$

The formula to perform the inverse mapping is denoted as

(2) $${Log}_{P}\left(S_{i}\right)=P^{\frac{1}{2}}\mathrm{Log}(P^{-\frac{1}{2}}P_{i}P^{-\frac{1}{2}})\;P^{\frac{1}{2}}$$

Once the tangent space vectors have been extracted, we may use the Principal Component Analysis (PCA) and ANOVA methods as a variable selection strategy to lower the space dimension and alleviate the curse of dimensionality.

### 3.4. EEG Classification

In this study, we rigorously tested multiple ensemble learning algorithms, including Random Forests (RF), Extremely Randomized Trees (ERT), Gradient Tree Boosting (GTB), AdaBoost, and Voting, for their ability to recognize APPD mental states. A modified version of the 5-fold cross-validation process based on stratification was used to assess the quality of the proposed approach.

Five-fold cross-validation is a commonly employed technique in machine learning to assess the performance of algorithms. The method involves dividing the original dataset into five equal-sized subsets, referred to as folds. In turn, each fold serves as the validation data once while the remaining four folds are utilized as training data. This process is repeated five times, with each fold being used exactly once as the validation data. The performance of the algorithm is then evaluated based on the average of the results obtained from the five trials.

This approach to evaluating performance provides a more reliable estimate compared to a single train/test split. This is due to the reduction of variance in performance estimates and the assurance that all data are utilized for both training and testing.

RF: In 2001, L. Breiman presented the Random Forest algorithm as a general-purpose classification and regression technique, and it has since seen tremendous success. The method has been shown to be effective in situations when there are more variables than observations, as it mixes multiple randomized decision trees and averages their predictions. It can be scaled up to address complex issues, customized to meet the needs of a wide range of ad hoc learning projects, and designed to yield metrics of varying significance. The entropy function was used as a metric of split quality in our work, with the number of estimators fixed at 200.

ERT: It is a classifier that works in a way that is similar to RF, but with a slight twist: it introduces randomization to the training process. Each tree in ExtraTrees’s multiple trees is trained independently using the entire dataset used for the classification. The optimum branching at a node is determined by considering a subset of all features, much like the Random Decision Forest. Each feature has a single threshold picked at random rather than multiple, less optimal ones. In our research, we used a total of 200 estimators and the entropy function to evaluate split quality.

GTB: It provides a prediction model in the shape of a collection of weak prediction models, most often decision trees. GTB is the name of the resulting procedure when a decision tree is the weak learner. The method extends the boosting algorithm to any loss function that can be differentiated. In our study, split quality was assessed using the ‘friedman_mse’ function and a total of 100 estimators.

AdaBoost: The statistical classification meta-algorithm known as Adaptive Boosting was developed by Yoav Freund and Robert Schapire in 1995. Its performance can be enhanced by combining it with a variety of different learning methods. This method creates a model in which each piece of information is given the same amount of consideration. Incorrectly labelled points are thus given more weight. After this new model is created, the points with greater weights will be given more consideration. A model will be trained repeatedly until a reduced error is received. Because of its rapid convergence to a smaller test error after fewer boosting iterations, the ‘SAMME.R’ method was chosen in our research.

The hybrid model (Voting): The goal is to predict class labels using a majority vote or the average projected probability (soft vote) based on the results of a collection of machine learning classifiers that are conceptually distinct from one another. A classifier like this can help even out the performance of a group of otherwise comparable models. Based on the outcomes of RF, ERT, and GTB, we used the average projected probability to make predictions about class labels.

### 3.5. Performance Metrics

Several indicators are used to determine the reliability of our findings. The Confusion Matrix is the most important criterion for evaluating our classification models. Metrics like a model’s accuracy, precision, and recall are also crucial for understanding how well it actually performs. True positive (TP), false positive (FP), true negative (TN), and false positive (FN) are the four concepts used in the metrics. In greater detail, these metrics are described as follows:

Accuracy: It is the proportion of accurately predicted classes achieved by the model. The formal definition is as follows:

(3) $$Accuracy=\frac{TP+TN}{TP+FP+TN+FN}$$

Precision: It can be defined as the percentage of positive observations that were successfully anticipated relative to the total number of positive observations that were predicted. The formal definition is as follows:

(4) $$Precision=\frac{TP}{TP+FP}$$

Recall: It can be calculated by dividing the number of accurately anticipated positive observations by the total number of observations in the actual class. The formal definition is as follows:

(5) $$Recall=\frac{TP}{TP+FN}$$

F1-score: It is the weighted average of Precision and Recall. The formal definition is as follows:

(6) $$F1-score=2\times \frac{Precision\times Recall}{Precision+Recall}$$

## 4. Results and Discussion

In this study, a multimodal approach was proposed to identify attention-related pilot performance-limiting states based on heterogeneous EEG data. We employed an automated preprocessing pipeline to clean the EEG data by either removing or repairing corrupted epochs. We employed an extraction and selection methodology based on Riemannian geometry analysis to obtain meaningful features from the cleaned data. Using these extracted features, we trained a hybrid ensemble learning model in addition to four other ensemble learning models to detect APPD states.

### 4.1. EEG Signal Analysis

**Figure 5:**
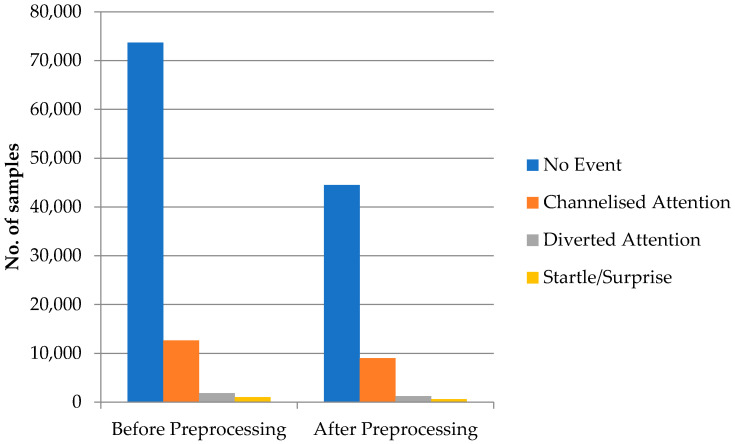
The size of the dataset before and after preprocessing the dataset.

This section presents and discusses the results of employing the automated preprocessing pipeline. Figure 5 reveals the size of the dataset before and after preprocessing the dataset.

We observed that the proposed pipeline identified and discarded a total of 33,786 contaminated epochs in the dataset; to be precise, 29,175 epochs from the NE class, 3632 epochs from the CA class, 598 from the DA class, and 381 epochs from the SS class were dropped from the dataset, as they were considered artefacts.

The proposed EEG preprocessing pipeline aims to improve the quality of EEG data by removing artifacts and other sources of noise, ultimately leading to more accurate and reliable results in downstream analyses. The employed pipeline removed 33,786 out of 89,198 epochs were recorded, resulting in a final dataset of 55,412 epochs. While some may argue that removing such a large number of epochs may lead to a loss of valuable data, it is important to consider the rationale behind the preprocessing steps and the impact they have on the quality of the remaining epochs.

**Figure 6:**
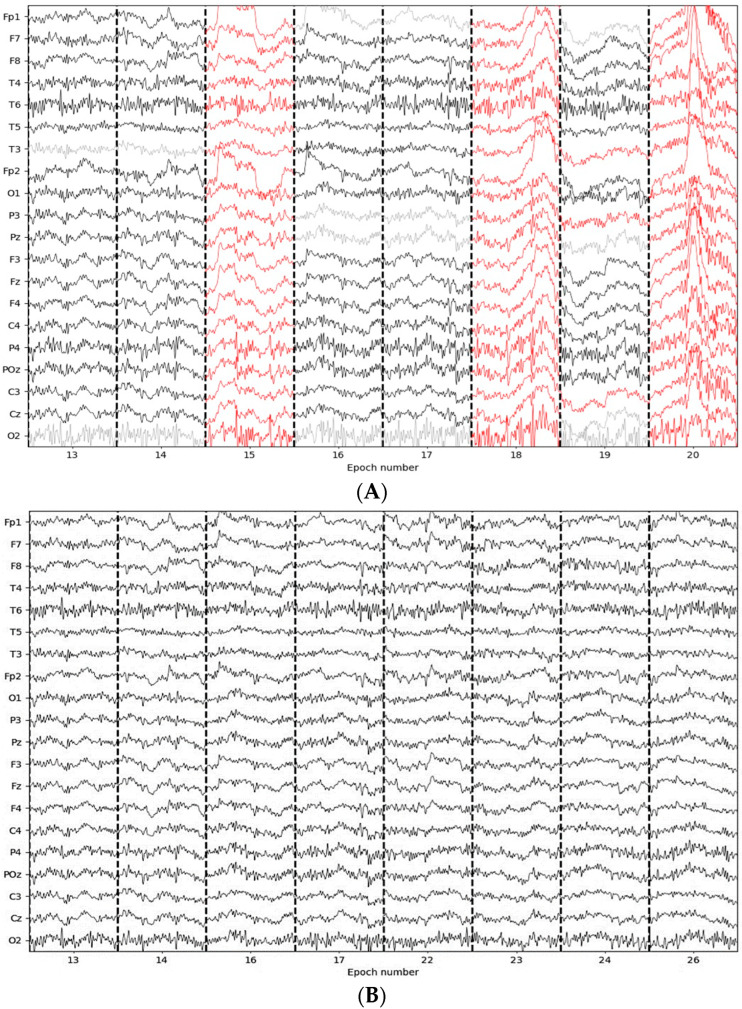
An eight-epoch example of the EEG signals before and after preprocessing.

While visually inspecting the discarded epochs, we observed that the epochs were contaminated by physiological artefacts, such as muscle tension and clenching of the jaw, and non-physiological/technical artifacts, such as body movements and powerline interference. As an illustration, Figure 6A depicts an eight-epoch window of the original EEG data, whereas Figure 6B depicts an eight-epoch window of the EEG data that have been preprocessed using the preprocessing pipeline. Figure 6A reveals that ocular activity artefacts, such as blinks and lateral eye movements, were spotted and color-coded as red in epochs 15, 18, and 20. These three epochs were deleted in addition to epochs 19, 21, and 25, as indicated in Figure 6B. We also noticed that some epochs, epoch 16 for instance, were repaired.

Based on the results presented, the EEG preprocessing pipeline appears to be effective in improving the quality of the EEG data. The visual comparison of the EEG signal before and after preprocessing indicates a reduction in noise and artifacts, resulting in a cleaner and more consistent signal.

The use of Autoreject for artifact rejection and correction, followed by eye-related artefact removal, and a second stage of Autoreject for further correction, provides a comprehensive approach to minimizing the impact of artefacts on the EEG signal. The use of these methods in combination is likely to capture a wide range of artefacts and improve the overall quality of the data.

The effectiveness of the pipeline is also supported by the quantitative analysis of the EEG data. For example, the reduction in the number of epochs removed after preprocessing may indicate that the pipeline was successful in identifying and removing a significant proportion of the artifacts. Furthermore, the comparison of the EEG data before and after preprocessing may provide evidence of the improvements made in the EEG data quality.

However, it is important to note that the effectiveness of the pipeline may depend on various factors, such as the quality of the initial EEG data and the parameters used for each stage of the pipeline. Therefore, a careful evaluation of the resulting EEG data and the quality of the analysis should be conducted to determine the overall effectiveness of the pipeline.

In addition, while the use of automated methods for artefact detection and correction can provide several advantages, such as consistency and efficiency, they may not capture all sources of noise and artifacts. Therefore, it may be beneficial to supplement the automated methods with visual inspection, especially in cases where subtle sources of noise may be present.

**Figure 7:**
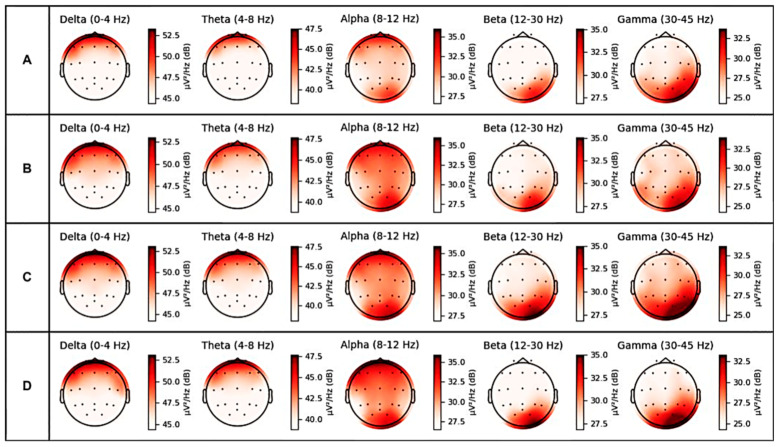
Spectral power topography during APPD mental states, namely (**A**) NE, (**B**) SS, (**C**) CA, and (**D**) DA.

We also report the spectral power analysis of one pilot while performing the high-fidelity motion-based flight simulator experiment to examine the overall activity level of the brain at different frequencies. Figure 7 illustrates the spectral power topography during APPD mental states, namely (A) NE, (B) SS, (C) CA, and (D) DA. The power spectral density was computed for each frequency band (delta (0–4 Hz), theta (4–8 Hz), alpha (8–12 Hz), beta (12–30 Hz), and gamma (30–45 Hz)).

In all frequency bands, we commonly found an increase mean power of the CA, DA, and SS states compared to the NE state. We also observed a lower frequency power increase in all frequency band ranges during the SS state. For the delta activity, the highest mean spectral power was located in the frontal lobe during the CA and DA states. For the theta and alpha activity, the highest spectral power was observed in the frontal lobe for theta activity (max: 47.5 dB) and in the frontal and occipital lobes for alpha activity (max: 36.7 dB) during the DA state. Theta oscillations have been linked to mental states of relaxation and drowsiness, while alpha oscillations have been associated with decreased cognitive engagement and mind-wandering. For the beta (max: 33.3 dB) and gamma activity (max: 33 dB), the highest spectral power was observed in the occipital lobe during the CA state. Both beta and gamma oscillations have been connected to engaged cognitive processing, including perception and memory, while beta oscillations have been associated with focused attention and concentration.

Spectral power analysis is a well-established method for analyzing EEG data that has been used in many studies to investigate the spectral properties of the EEG signal. In our study, we used spectral power analysis to visualize the topography of EEG activity during four different mental states—CA, DA, SS, NE. By calculating the power spectral density of the EEG signal in different frequency bands, we were able to obtain topographical maps that showed the distribution of power across the scalp. These maps provided a global view of the EEG patterns that were associated with each mental state, and they allowed us to identify the scalp regions that exhibited the strongest or weakest power in different frequency bands. This information was useful in identifying patterns of EEG activity that were associated with each mental state, and in validating the results of our subsequent classification analysis. Thus, the use of spectral power analysis was essential to achieving the primary objective of our study, which was to gain a better understanding of the EEG patterns underlying the four mental states.

### 4.2. Evaluation of Machine Learning Models

Five ensemble learning models, namely RF, ERT, GTB, AdaBoost, and Voting, were trained with tangent space features generated from cleaned EEG data using the 5-fold cross-validation technique. First, we estimated the spatial covariance matrices from the cleaned EEG data and obtained a set of SPD matrices of shapes (48, 48). Each matrix was vectorized, obtaining 1176 tangent space features, which were then projected to a lower dimensional space using PCA. In Table 1, we show the performances of the employed ensemble learning models. We considered the macro average of the evaluation metrics Accuracy, Recall, Precision, and F1-score. We also show the standard error, which we calculated based on the F1-score metric for each class because we trained the models using the 5-fold cross-validation technique.

**Figure 8:**
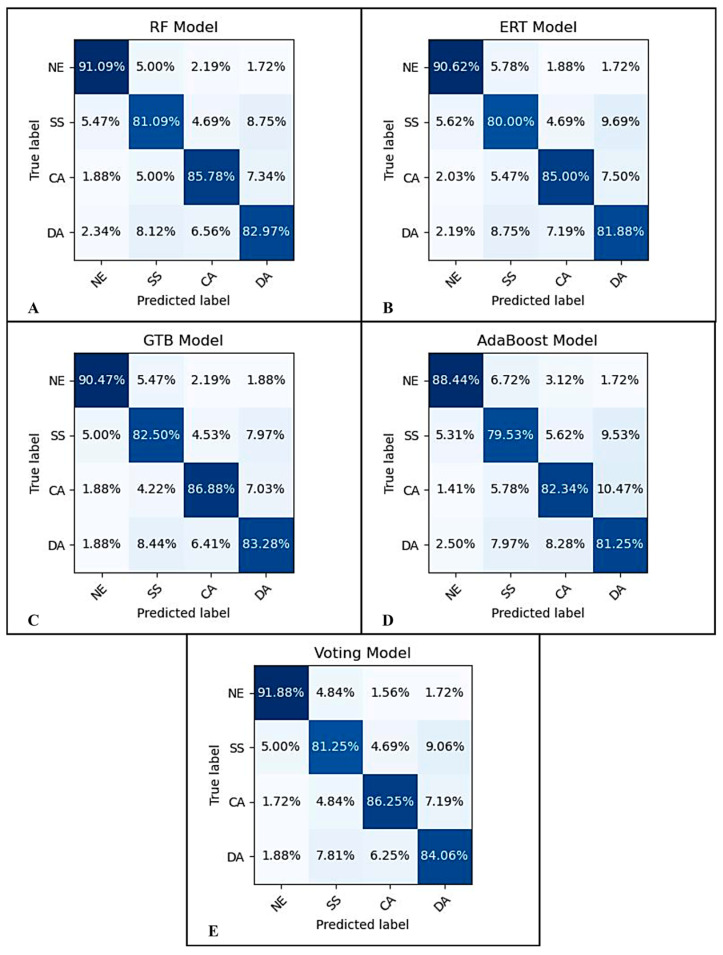
The confusion matrix for the 5-fold cross-validation results. The RF model’s confusion matrix is shown in (**A**); the ERT in (**B**), GTB in (**C**), AdaBoost in (**D**), and Voting in (**E**).

To provide thorough analysis, the degree of confusion generated by each model was computed. The confusion matrix for the 5-fold cross-validation results using the RF classifier is shown in Figure 8A; the ERT was employed in (B), GTB in (C), AdaBoost in (D), and Voting in (E). The values of the diagonal elements represent the percentage of correctly predicted classes.

Based on the data from Table 1, we observed that all five models provided good detection performances. The best accuracy performance achieved was 86%, which was achieved by the RF, GTB, and Voting models, followed by AdaBoost (84%) and ERT (83%). The same trend can be seen across different metrics, including precision, recall, and F1-score. We believe the reason why ERT did not perform as well as the RF model, although both algorithms are based on the bagging or bootstrap aggregation technique, is because of the randomness in the way splits are computed; while the most discriminative thresholds are picked as the splitting rule in RF, thresholds in ERT are drawn at random, which slightly increased biasness in the model. Similarly, we also observed a slight difference in the performances of GTB and AdaBoost, even though both algorithms are based on the boosting technique. We suspect the reason of the increase in GTB model performance is due to the use of the log loss function, which is more robust to mislabeled examples in the dataset; unlike GTB, the AdaBoost algorithm uses the exponential loss function.

Figure 8 further shows that all models made accurate classification predictions. The NE mental state was predicted by all five models to be the easiest to distinguish, with an accuracy performance range of 88.44–91.88%, followed by the CA with a range of 82.34–86.88%. It was also discovered that, across all five models, DA was the third best at recognizing class with an accuracy performance of 81.25–84.06%, while SS was the worst at recognizing class with an accuracy performance of 79.53–82.50%. Nevertheless, these performance levels can be enhanced if the dataset is more cohesive. With regards to predicting NE and DA states, the Voting classifier performed best, whereas the GTB classifier performed best with regards to predicting CA and SS states.

The use of ensemble models has become increasingly popular in machine learning due to their ability to leverage the strengths of different models to improve performance. In this study, we compared the performance of several popular ensemble models, including RF, ERT, GTB, and AdaBoost, with a hybrid ensemble model. The results showed that the hybrid ensemble model outperformed ERT and AdaBoost and achieved comparable performance to RF and GTB. One of the key advantages of the hybrid ensemble model is its flexibility. By combining different models, the hybrid ensemble approach can handle various types of data and tasks, making it a versatile option for different applications. In contrast, the other models tested in this study were each based on a single algorithm, limiting their flexibility to some extent. Another advantage is its improved generalization ability. The use of a combination of models in the hybrid ensemble approach can help to mitigate the risk of overfitting. This can lead to more accurate predictions on new, unseen data, making the hybrid ensemble model a promising approach for practical applications.

Several studies have investigated the classification of mental states using EEG data. However, some of these studies did not make their dataset publicly accessible, did not achieve clear or consistent results, employed different sensors and conventional preprocessing techniques, or did not classify the same number of mental states. In order to compare the results of our multimodal approach with other studies, we evaluated our approach in the context of studies that have used the same dataset.

Harrivel et al. [35] implemented a broad suite of sensors to classify pilot mental states. Although this study provided initial insights into the use of physiological signals to measure attention in aviation, their datasets were limited in size. In addition, their results were not conclusive because they were based on only one pilot. Harrivel et al. [34], on the other hand, considered a larger sample size and employed multiple sensors, including EEG, ECG, GSR, and respiration. However, the study relied on spectral power features and did not classify four mental states. Moreover, the results were not as good as in our study, likely due to the limited classification capabilities of spectral power features. Similarly, [20] considered a larger sample size of 18 users but did not clean their data from artifacts and merged three mental states into one called the event state. The lack of artifact removal may have contributed to unclear results, and the use of different metrics limited comparison with our study.

We also evaluated our approach in the context of studies that have used a different dataset. For example, Han et al. [25], proposed a multimodal deep learning network to classify four mental states (distraction, baseline, workload, and fatigue) using a dataset of eight pilots. The authors employed conventional preprocessing techniques, including filtering and ICA for removing eye-related artifacts. They also extracted PSD features from the EEG signals and provided three topographic maps as inputs to a CNN model. In addition, the authors employed ECG, GSR, and respiration signals as inputs to an LSTM network. However, the dataset used by Han et al. was not a publicly accessible dataset, unlike our study and studies [20,29,34,35], which were all publicly available. While their results were promising, the small sample size and lack of a public dataset may limit the generalizability of the findings. In addition, our approach achieved an accuracy of 86% in detecting mental states, which is a substantial improvement over Han et al. study’s performance of 77.7%. Hernández-Sabaté et al. [43] developed a CNN model to classify different mental workloads of pilots using EEG signals. Although they made their dataset publicly available, they divided a signal state to multiple states.

In comparison to our previous study [29], where we evaluated the impact of different preprocessing techniques on the performance of ML algorithms for classifying pilots’ mental states, the current study represents a significant improvement in mental state detection.

In this study, we developed a novel multimodal approach that includes advanced automated preprocessing techniques, Riemannian geometry-based feature extraction, and a hybrid ensemble learning technique that combines the results of several machine learning classifiers. The use of Riemannian geometry analysis for feature extraction and the hybrid ensemble learning technique outperforms traditional approaches and shows the importance of advanced techniques in improving the accuracy of mental state detection. Our approach is the first of its kind because it combines advanced techniques proposed in three different fields: Autoreject, from the neuroscience field for data preprocessing; Tangent space mapping, from BCI for feature extraction; and hybrid ensemble learning artificial intelligence for pilot’s mental states classification.

This study can have significant implications for improving pilots’ performance and safety in the aviation industry. Our approach has the potential to benefit several sectors within the aviation industry. One important application is in pilot training and performance evaluation. By accurately characterizing pilot mental states using EEG data, the proposed approach can be used to identify areas where pilots may need additional training or support, and to evaluate the effectiveness of training programs in improving cognitive performance.

Another potential application is in aviation safety, particularly in identifying potential safety hazards related to pilot mental states. By providing a detailed and accurate characterization of pilot mental states during flight, the proposed approach can help identify situations where pilots may be at higher risk of making errors or experiencing cognitive overload, allowing for proactive interventions to be taken to prevent accidents and improve safety.

Additionally, our approach has the potential to improve human–machine interaction in the aviation industry. By using EEG data to monitor pilot mental states, future BCI systems can be developed that are better able to adapt to the cognitive state of the pilot, improving the efficiency and safety of the aviation system as a whole.

Overall, the potential applications of our approach are diverse and have the potential to make a significant contribution to the aviation industry by improving safety, training, and human–machine interaction.

## 5. Conclusions

We conducted an exploratory investigation using uncontaminated EEG data and ensemble learning algorithms to characterize the pilot’s mental states (i.e., channelized attention, diverted attention, startle/surprise, and normal). We also demonstrated how the pilot’s varied mental states impacted physiological indicators. With the goal of identifying the neural signal related to cognitive processes reflective of brain activity while disregarding the other artefacts and extracting significant information, we proposed a feasible approach for automatically preprocessing EEG data. In order to proceed to the classification phase, the processed data underwent feature extraction, during which spatial covariance matrices were calculated and subsequently mapped onto the Riemannian tangent space. Four ensemble learning models, namely RF, ERT, GTB, and AdaBoost, and a hybrid ensemble model were trained using tangent space vectors.

Based on the findings, it was clear that the proposed method successfully identified artifacts in the EEG epochs and either fixed or discarded them automatically. In addition, the results indicated the viability of implementing EEG-based BCI systems, such as tangent space mapping, to characterize the pilot’s mental states. According to the findings of the pilot’s mental states detection investigation, we observe that the RF, GTB, and the hybrid ensemble models are the best at predicting NE, CA, SS, and DA states, with an accuracy rate of 86%.

The innovative nature of this study lies in its combination of advanced automated preprocessing techniques, Riemannian geometry-based feature extraction, and ensemble learning models, which, together, provide a detailed and accurate characterization of pilot mental states, ultimately leading to a safer and more efficient aviation system.

The models’ performances will be further refined, and the training dataset will be enlarged, in subsequent work. We also aim to apply the aforementioned approach to a broad range of machine learning and deep learning models. In further studies, we can also investigate the possibility of extracting other meaningful features.

## Figures and Tables

**Table 1:** Ensemble learning models’ performances.

Methods	Mental Class	Accuracy (%)	Precision (%)	Recall (%)	F1-Score (%)	Standard Error
RF	NE		91	92	91	0.010
	SS		82	81	82	0.009
	CA		87	86	87	0.013
	DA		82	83	83	0.011
	Macro average	86	86	86	86	
ERT	NE		90	91	90	0.011
	SS		80	80	80	0.016
	CA		86	85	86	0.010
	DA		81	82	82	0.012
	Macro average	84	84	84	84	
GTB	NE		91	90	91	0.016
	SS		82	82	82	0.009
	CA		87	87	87	0.012
	DA		83	84	83	0.011
	Macro average	86	86	86	86	
AdaBoost	NE		91	88	89	0.009
	SS		80	80	80	0.007
	CA		83	82	83	0.010
	DA		79	81	80	0.023
	Macro average	83	83	83	83	
Voting	NE		91	92	92	0.013
	SS		82	82	82	0.009
	CA		87	86	87	0.012
	DA		83	84	83	0.013
	Macro average	86	86	86	86	

**Figure A1:**
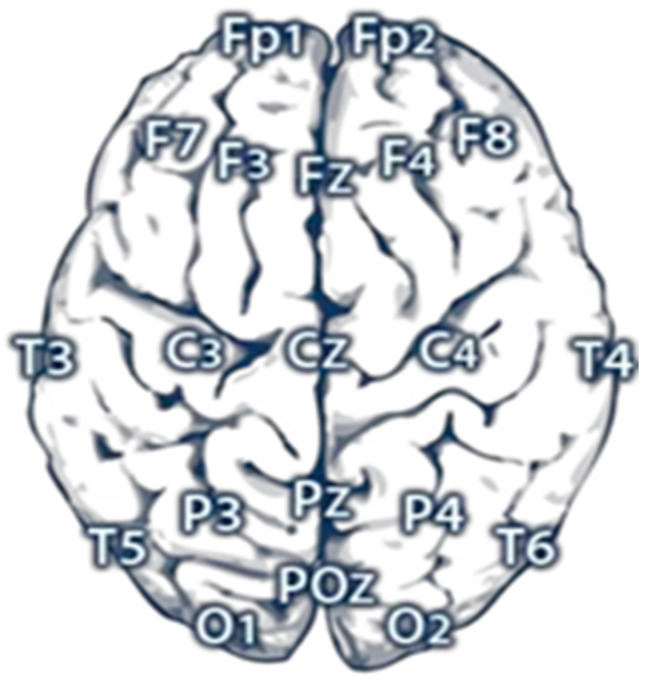
EEG electrode names and locations.

## Data Availability

The source code and the data used for the experiments are made freely available under the MIT License and can be downloaded from https://doi.org/10.17862/cranfield.rd.22232062 (accessed on 1 July 2023).

## Appendix A

### Appendix A.1. Advanced Brain Monitoring X24 EEG Headset

The X24 EEG headset was employed to gather the EEG dataset. This headset offers a wireless option for acquiring and recording EEG signals without the need for scalp abrasion. It is equipped with 20 electrodes arranged in the standard 10–20 format and one additional electrode, POz, as shown in Figure A1. These electrodes are located at specific locations on the head, such as Fz, Cz, Pz, F3, F4, C3, C4, P3, P4, O1, O2, T5, T3, F7, Fp1, Fp2, F8, T4, T6, and Linked Mastoids. The wireless technology allows for freedom of movement for the user during data collection and display in real-time. The headset collects EEG signals from the sensors on the participant and processes the signals through analog-to-digital conversion, encoding, formatting, and transmission. It operates at a sample rate of 256 Hz and uses the system’s bi-directional capabilities to check scalp-electrode impedance and monitor battery capacity in the X24 Headset. Figure A1 illustrates the names and locations of the electrodes on the EEG sensor.

### Appendix A.2. Flight Simulator

The dataset was obtained from 18 commercial aviation pilots who participated in a research flight deck simulation at NASA Langley Research Center. The flight deck, which is known as the cockpit motion facility, is an all-glass reconfigurable cockpit that is equipped with a programmable sidestick and pedal control inceptors. The simulator, which can operate in both motion-based and fixed-base modes, is designed to provide a high-fidelity, full-systems flight experience for pilots. It is used to evaluate and improve research concepts related to flight crew operations, covering everything from engine startup to engine shutdown.
